# Exogenous Carbon Compounds Modulate Tomato Root Development

**DOI:** 10.3390/plants9070837

**Published:** 2020-07-03

**Authors:** Ana Isabel González-Hernández, Loredana Scalschi, Pilar García-Agustín, Gemma Camañes

**Affiliations:** Grupo de Bioquímica y Biotecnología, Área de Fisiología Vegetal, Departamento de Ciencias Agrarias y del Medio Natural, ESTCE, Universitat Jaume I, 12071 Castellón, Spain; scalschi@uji.es (L.S.); garciap@uji.es (P.G.-A.)

**Keywords:** *Solanum lycopersicum*, root development, N metabolism, sugars, 2-oxoglutarate

## Abstract

NO_3_^−^ is not only a nutrient, but also a signaling compound that plays an important role in several plant processes, like root development. The present study aimed to investigate the effect of three different exogenous C compounds (sucrose, glucose, 2-oxoglutarate) added to NO_3_^−^ nutrition on C/N, auxin and antioxidant metabolisms in 10-day-old tomato seedlings. Sucrose and glucose supplementation enhanced primary root (PR) length, lateral root number and root density, while 2-oxoglutarate negatively affected them. This phenomenon was accompanied by a slight increase in *NRT2.1* and *GS1* gene expression, together with an increase in *LAX2* and *LAX3* and a decrease in *LAX4* in the roots growing under sucrose and glucose sources. The addition of 2-oxoglutarate enhanced the expression of *NiR*, *GDH*, *PEPC1*, *LAX1*, *LAX3* and the antioxidant gene *SOD Cl*. Taken together, these findings contribute to a better understanding of how these C sources can modulate N uptake and C/N, auxin and antioxidant gene expression, which could be useful for improving nitrogen use efficiency.

## 1. Introduction

Plants need to coordinate organ formation and growth in response to environmental constraints like nutrient status. Nitrogen (N) is a limiting factor for plant growth as it takes part in nucleic acids, amino acids and phytohormones [1]. N can be acquired by roots as nitrate (NO_3_^−^), ammonium (NH_4_^+^), urea, amino acids and peptides, but NO_3_^−^ is one of the commonest N forms available to plants in aerobic and high pH soils [2,3]. This anion is not only a nutrient, but also a signaling compound that orchestrates the root system architecture, among other physiological processes [4]. It is known that NO_3_^−^ deficiency increases root density and root hair length in spinach and tomato [5]. A moderate NO_3_^−^ supply induces root growth, whereas excess of NO_3_^−^ leads to inhibition in *Arabidopsis thaliana* [6,7]. Several studies have previously shown that root growth parameters are sensitive to carbon (C) availability, which confirms the role of sugars in nutrition and signaling processes [8,9]. It has been reported how the addition of glucose (Gluc) induces root parameters in a concentration-dependent way [10]. Moreover, incremented lateral root (LR) development has been observed in *Arabidopsis* mutants with defects in the leaf cuticle, which took up more sucrose (Suc) from the medium under high NO_3_^−^ conditions [11]. Furthermore, exogenous spraying application of trehalose promotes *Nicotiana tabacum* plant growth under N-limiting conditions by up-regulating N assimilation enzyme activities [12].

Plants have developed a specialized mechanism to uptake and assimilate N compounds [13]. NO_3_^−^ is taken up via low- and high-affinity NO_3_^−^ localized transporters, the so-called Nitrate Transporters (NRTs). There are two families of NRT (NRT1 and NRT2) and only five genes have been identified in tomato: *NRT1.1*, *NRT1.2*, *NRT2.1*, *NRT2.2* and *NRT2.3* [14,15,16,17,18]. NO_3_^−^ is reduced to NO_2_^−^ and NH_4_^+^ by nitrate reductase (NR) and nitrite reductase (NiR), respectively. NH_4_^+^ is assimilated in N-containing compounds through a set of enzymatic reactions. The starting point is the incorporation into Gln and Glu by enzyme glutamine synthetase (GS)/glutamine-oxoglutarate aminotransferase (GOGAT) [19]. Asparagine synthetase (ASN1) enzyme catalyzes Asp and Gln transformation into Asn which plays a role in N transport and storage [20]. In addition, Glu could be converted into either of the other amino acids or 2-oxoglutarate by the glutamate dehydrogenase (GDH) enzyme [21]. This last reaction indicates a link between N and C metabolism, and it is noteworthy that the genes of the organic acid pathway, phosphoenolpyruvate carboxylase (*PEPC*) and pyruvate kinase (*PK*), provide C skeletons, which are required for the NO_3_^−^ assimilation process and amino acid biosynthesis [22]. Therefore, it can be clearly deduced that N assimilation is linked with C assimilation [23], as C- and N-derivate molecules can act as signalling compounds to regulate the expression of several genes and different developmental processes that bring about various changes in the plant phenotype [24]. In addition, sugar modulates root growth and nitrate uptake because seedlings grown at high C/N ratio repress lateral root initiation which seems to depend on auxin location [25,26].

Auxins act as shoot-root signals. They are transported by a set of influx and efflux transporters, which are auxin influx carriers (AUX/LAX) and pin-formed auxin efflux (PIN), respectively [27,28,29,30]. In tomato, *AUX1/LAX* and PIN gene families respectively contain five (*LAX1-LAX5*) and ten (*PIN1-PIN10*) members [31], each of which seems to play a role in different plant tissues. For example, these authors [31] have suggested that *PIN3* and *PIN4* are involved in the determination of tomato plant architecture. Moreover, auxin-efflux activity also depends on the ATP-binding cassette (*ABC*) superfamily [32]. Auxin is a regulator of root development in response to NO_3_^−^ nutrition [29,33] and NO_3_^−^ availability can modulate the auxin concentration in root tissue, as a high NO_3_^−^ concentration might inhibit auxin biosynthesis [34]. A molecular link between auxin and reactive oxygen species (ROS) in plant development has been recently suggested because auxin regulates the induction of ROS-related genes, which affect *Arabidopsis* and tomato root development [35,36,37]. Traditionally, ROS have been related to oxidative stress, but are currently considered to be plant growth regulators [38]. The commonest ROS is H_2_O_2_, which seems to play a role in LR development [39], although H_2_O_2_ can also act as an inhibitor of photosynthesis. Therefore, it is also important for ROS-scavenging enzymes, such as glutathione synthetase (GSH) or superoxide dismutase (SOD), to maintain ROS levels [40]. GSH-deficient mutants display defects in LR formation [41,42], and MnSOD-deficient mutants undergo both retarded root growth and an alteration to mitochondrial redox balance [43].

Altogether, these scenarios led us to study tomato root development and N transcriptional levels under NO_3_^−^ nutrition (NN) together with the addition of three C compounds, Suc, Gluc or 2-oxoglutarate (2-OG), to improve nitrogen use efficiency (NUE) and root adaptive responses in the first seedling growth stages. We further discuss auxin transporters and antioxidant gene expression responses under the aforementioned conditions.

## 2. Results

### 2.1. Carbon Sources Effect on Root and Shoot Development

**Figure 1:**
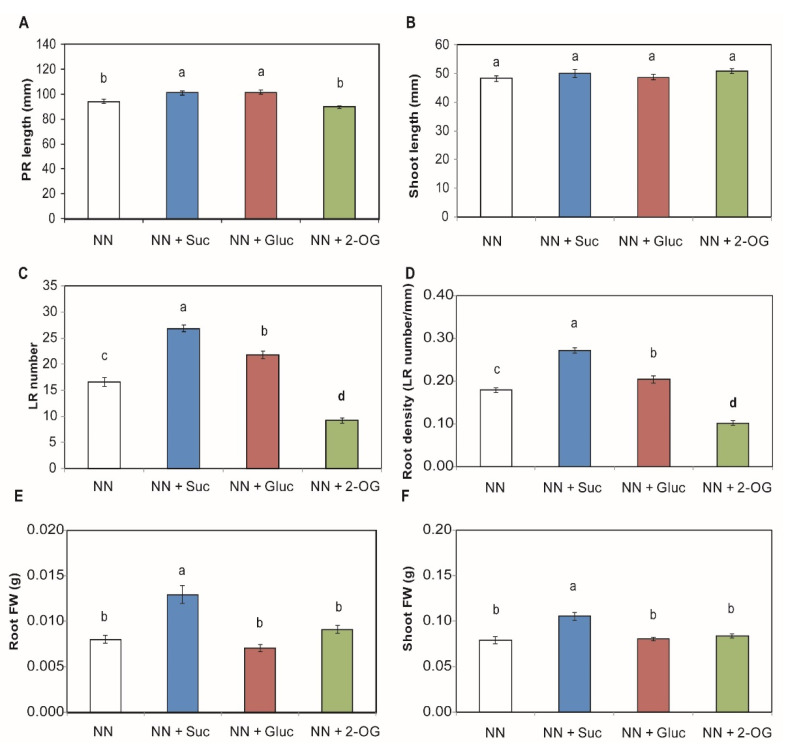
Root and shoot development is modified by adding exogenous C compounds. Seedlings of tomato plants were grown with the addition of sucrose (Suc), glucose (Gluc) or 2-oxoglutarate (2-OG). Primary root (PR) length (**A**), shoot length (**B**), lateral root (LR) number (**C**) were measured. Root density was calculated as the LR number divided by the PR length (**D**). Root fresh weight (FW) (**E**) and shoot FW (**F**) were measured. The data shown are the mean of at least three independent experiments ± standard error (SE). Distinct letters indicate statistically significant differences among treatments as determined by Tukey honest significant difference (HSD) (*p* < 0.05).

To study the root and shoot development in response to different exogenous C sources, Suc, Gluc and 2-OG were added to NN medium to grow tomato seedlings. The addition of sugars (Suc and Gluc) increases primary root (PR) development. However, the root growth did not change when organic acid 2-OG was applied (Figure 1A). Sugar supply, nor 2-OG addition, produces changes in shoot development (Figure 1B). It is noteworthy that the use of both sugars significantly increased the LR number, Suc and Gluc led to a 1.6-fold and 1.3-fold increase, respectively, compared to the tomato seedlings grown in NN medium. However, 2-OG supply brought about a LR number reduction of 55% (Figure 1C), so the addition of exogenous C sources affected root density (Figure 1D). Finally, it should be pointed out that among the different exogenous C sources added to NN medium, sucrose was the only C compound capable of significantly increasing root and shoot fresh weight (FW) (Figure 1E,F).

### 2.2. Carbon Sources Effect on NO_3_^−^ Assimilation

**Figure 2:**
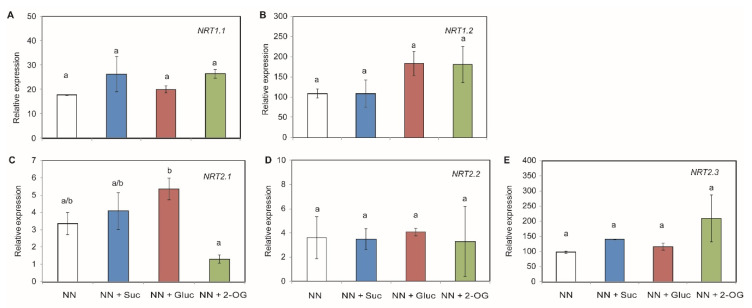
Changes in the relative gene expression of NO_3_^−^ transporters in tomato root tissue. Seedlings of tomato plants were grown with addition of Suc, Gluc or 2-OG as C sources. The studied genes were: *NRT1.1* (**A**), *NRT1.2* (**B**), *NRT2.1* (**C**), *NRT2.2* (**D**) and *NRT2.3* (**E**). The data shown are the mean of at least three independent experiments ± standard error (SE). Distinct letters indicate statistically significant differences among treatments as determined by Tukey HSD (*p* < 0.05).

NO_3_^−^ uptake by roots is mediated by the nitrate transporter system encoded by the *NRT1* and *NRT2* gene families. We observed that the *NRT1* genes were not affected by adding exogenous C sources (Figure 2A,B). With the *NRT2* gene family, *NRT2.1* gene expression was moderately repressed when 2-OG was present in the medium. However, its expression was slightly—but not significantly—induced in the presence of both Suc and Gluc (Figure 2C). The expression of genes *NRT2.2* and *NRT2.3* was not affected by the exogenous addition of the tested C sources (Figure 2D,E).

**Figure 3:**
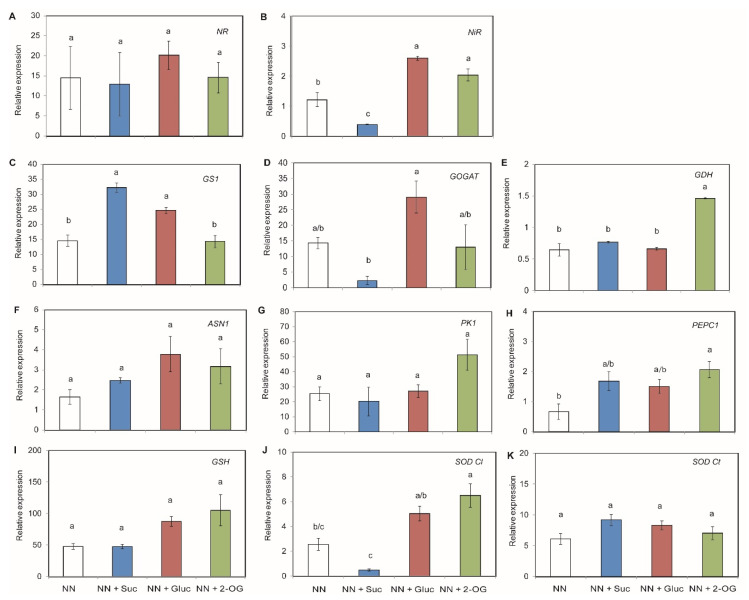
Changes in relative gene expression of NO_3_^−^ assimilation genes in tomato root tissue. Seedlings of tomato plants were grown with addition of Suc, Gluc or 2-OG. The studied genes were: *NR* (**A**), *NiR* (**B**)**,** *GS1* (**C**), *GOGAT* (**D**), *GDH* (**E**), *ASN1* (**F**), *PK1* (**G**), *PEPC1* (**H**); *GSH* (**I**), *SOD Cl* (**J**) and *SOD Ct* (**K**). The data shown are the mean of at least three independent experiments ± standard error (SE). Distinct letters indicate statistically significant differences among treatments as determined by Tukey HSD (*p* < 0.05).

Regarding NO_3_^−^ assimilation, the *NR* gene expression was not affected by the addition of exogenous C, while *NiR* gene expression was modified (Figure 3A,B). A strong repression of this gene was observed when Suc was present in the medium, but the relative expression was significantly up-regulated in response to adding Gluc and 2-OG (Figure 3B). *GS1* was strongly induced by Suc and Gluc (Figure 3C). Instead, *GOGAT* was more induced by Gluc addition and was repressed by Suc (Figure 3D). The conversion of 2-OG into Glu and vice versa was carried out by GDH. This gene expression increased, which correlated with 2-OG addition (Figure 3E). The ASN1 protein in charge of transferring the amino group of Glu to a molecule of Asp was not affected by C exogenous addition (Figure 3F). The phosphoenolpyruvate carboxylase 1 (*PEPC1*) gene, which codifies for the anaplerotic enzymes responsible for replenishing the TCA cycle, was induced when 2-OG was added to the medium (Figure 3H), while no significant differences were observed in pyruvate kinase 1 (*PK1*) gene expression when Suc, Gluc or 2-OG was present (Figure 3G). In order to study if the detoxification process would be affected by C source addition, the expression of the genes encoding detoxification enzymes was studied. The results showed that *GSH* expression was slightly up-regulated by 2-OG (Figure 3I). Moreover, the expression levels of the chloroplastic superoxide dismutase (*SOD Cl*) was only induced in 2-OG treatment (Figure 3J), while the cytoplasmic superoxide dismutase (*SOD Ct*) gene was not affected in any case (Figure 3K).

### 2.3. Auxin Transporters Gene Expression

**Figure 4:**
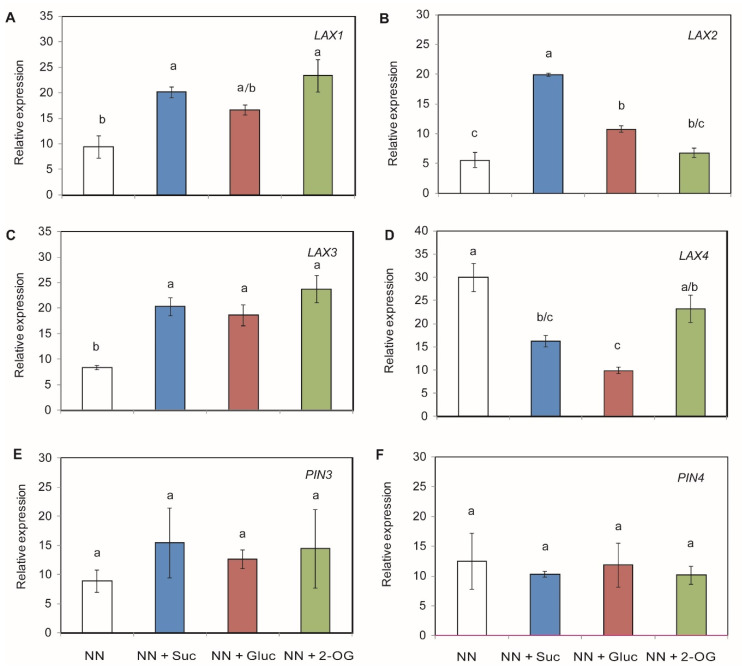
Changes in relative gene expression of auxin transporters in tomato root tissue. Seedlings of tomato plants were grown with addition of Suc, Gluc or 2-OG. The studied auxin transporters encoding genes were *LAX1* (**A**), *LAX2* (**B**), *LAX3* (**C**), *LAX4* (**D**), *PIN3* (**E**) and *PIN4* (**F**). The data shown are the mean of at least three independent experiments ± standard error (SE). Distinct letters indicate statistically significant differences among treatments as determined by Tukey HSD (*p* < 0.05).

Auxins are phytohormones involved in root development, which is regulated by auxin homeostasis and distribution through both auxin influx transporters (AUX/LAX) and auxin efflux carriers (PIN). To examine whether the changes observed in root development were related to the supplied C sources, the expression levels of the *LAX* and *PIN* family genes were measured. The expression of the *LAX1*, *LAX2* and *LAX3* genes was up-regulated when Suc and Gluc were added to NN medium, while 2-OG addition only up-regulated the expression of the *LAX1* and *LAX3* genes (Figure 4A–C). Interestingly, *LAX4* gene expression was down-regulated in the presence of Suc and Gluc sugars (Figure 4D). The relative gene expression levels of *PIN3* and *PIN4* were not affected by the exogenous addition of C sources (Figure 4E,F).

## 3. Discussion

Root growth is modulated depending on the N source and its availability in soil. It is already known that plants prefer to take up NO_3_^−^ and NH_4_^+^ forms from the soil. For this reason, the purpose of this work was to elucidate how the supply of different C sources to NO_3_^−^ growth medium could modulate the tomato seedling root architecture as well as the changes in N and C assimilation gene expression levels to examine how N and C metabolisms are involved in this process.

It has already been reported how NO_3_^−^ leads to different root development changes depending on the external NO_3_^−^ concentration. Previously published research describes how NO_3_^−^ stimulates LR elongation in *Arabidopsis thaliana*, but a NO_3_^−^ concentration higher than 10 mM can reduce LR branching and length [29]. A marked reduction in LR development in *Arabidopsis* plants has been demonstrated when they are exposed to a high sucrose-to-nitrogen (C:N) ratio [25,44]. In this study, as the tested sugar concentration was lower, we observed the opposite effect and our results showed an increase in PR length, LR number and root density when Suc and Gluc were added to NN medium. To support this result, previous works have described that seedlings supplemented with exogenous sugar display enhanced PR and LR development, which seems to be correlated with the level of sugar concentration in root tissue [45,46,47]. Moreover, the addition of 1% glucose to MS medium brings about changes in PR growth and LR in *Arabidopsis thaliana* seedlings [9]. However, in our work, Suc was the only sugar to increase shoot and root FW, perhaps because it is the main photosynthesis product and its exogenous addition might display enhanced photosynthetic activity [48]. We also observed that root parameters worsened or did not change with 2-OG supply compared to NN. Walch-Liu et al. [49] showed that exogenous glutamate application (first amino acid synthesized from 2-OG via GDH activity) inhibited PR growth. Moreover, this effect seemed to depend on the N source, as previously described in González-Hernández et al. [50], who revealed how adding a higher 2-OG concentration to NH_4_^+^ improved PR length, LR number, root density and fresh weight, but did not enhance shoot length. This effect seems to be related to the activation of amino acid biosynthesis to reduce NH_4_^+^ toxicity.

NO_3_^−^ uptake from soil is mediated by the *NRT1* and *NRT2* gene families in tomato plants [51]. This led us to study whether these transporters play a role in NO_3_^−^ and sugar-mediated responses. Under our experimental conditions, we observed almost no changes in the relative expression of these genes with the different C supplies except for *NRT2.1* relative expression. *NRT2.1* expression was slightly induced in the presence of Suc and Gluc. Previous studies have shown that *NRT2.1* expression is modulated under light and sugars (Suc and Gluc) by controlling root NO_3_^−^ uptake [52,53]. Our results indicated that *NRT2.1* gene expression was repressed when 2-OG was present in the medium. The same trend has been observed by Lejay et al. [51], who showed induction mediated by sugars, but no effect was revealed when organic acids were supplied. Once NO_3_^−^ uptake occurs, NO_3_^−^ assimilation is carried out by the activity of NR and NiR, where NH_4_^+^ is the obtained compound. It has long since been known that the expression of both genes depends on the day/night cycle [54,55], but this expression might also be influenced by other factors like the amount of reduced C available in roots. In our work, *NR* gene expression was not modified by exogenous C addition, while *NiR* gene expression was modified because a strong repression of this gene was observed when Suc was added to NN medium. The relative expression was significantly up-regulated in response to Gluc and 2-OG addition. In line with these results, Ali et al. [56] demonstrated an induction of NR and NiR in rice leaves treated with Glu and 2-OG. It is known that NH_4_^+^ accumulation in roots needs to be assimilated by the GS/GOGAT cycle [57,58]. Lin et al. [12] showed that by spraying tobacco leaves with trehalose, N assimilation was induced together with GS and GOGAT activities under N-deficient growing conditions. In our experimental system, *GS1* was strongly induced by Suc, and to a lesser extent by Gluc, while *GOGAT* seems to be induced by Gluc addition and repressed by Suc. This combined result suggests a marked NH_4_^+^ conversion for amino acid biosynthesis in Gluc- and Suc-treated seedlings, because sugars like Gluc had a stronger effect on N metabolism genes than N itself [59]. Thus, GDH converts 2-OG into Glu, and vice versa [19]. In line with this, we observed a slight increase in *GDH* gene expression in the seedlings supplied with 2-OG, but we did not find any changes in *ASN1* gene expression when 2-OG was added as it would appear to be induced by NH_4_^+^ nutrition, instead of NO_3_^−^ [50]. *PK1* (which codified for the anaplerotic enzymes responsible for replenishing the TCA cycle) was not induced when the C skeletons were added to the medium, while *PEPC1* gene expression was up-regulated, especially by the 2-OG source. Setién et al. [60] have previously described that enhanced PEPC activity is related to increased NH_4_^+^ assimilation due to the supply of C skeletons [60]. Therefore, the addition of the considered C compounds could enhance NO_3_^−^ assimilation and subsequent NH_4_^+^ assimilation in roots or leaf tissue.

In this work, we also studied the relative expression of auxin transporters as auxin plays an important role in root development. It is known that AUX1 and PINs auxin carriers are required for promoting LR initiation and primary root length [61,62]. Our results revealed that adding C sources to NN medium induced the LAX family genes (*LAX1*, *LAX2* and *LAX3*) but not the efflux carriers mediated by the PIN transporters family (*PIN3* and *PIN4*). Indeed, the expression of the *LAX1*, *LAX2* and *LAX3* genes was up-regulated when Suc and Gluc were added to NN medium, while the *LAX4* gene was down-regulated after both applications. Mishra et al. [9] and Sairanen et al. [63] have shown that Gluc up-regulates several genes related to auxin biosynthesis and transport machinery. Hence, the observed improvement in root growth parameters, such as a bigger LR number could be related to the downstream change in the expression of the auxin influx carriers’ gene, as previously described by Sun et al. [29] and Swarup et al. [30]. Revalska et al. [64] indicated that *LAX3* expression plays a role in root system modeling in *Medicago trunculata* plants. Our results demonstrated the up-regulation of *LAX1* and *LAX3* expression with 2-OG treatment. The mutant of both genes independently showed a reduction in LR formation, which indicates that they play an important role in LR development [65]. However, as the 2-OG-treated plants displayed the worst root phenotype, other pathways could have an antagonistic effect.

Finally, we studied the expression levels of detoxification genes *GSH*, *SOD Cl* and *SOD Ct* to check whether they would change with sugar supply. In our study, *GSH* expression was not up-regulated by any C skeleton additions. The expression levels of the *SOD Cl* gene were induced when 2-OG was supplied. It has been previously described how endogenous sugar changes can modify the expression of different antioxidant genes, such as superoxide dismutase [66]. Furthermore, MnSOD-deficient plants display stunted root growth which diminishes ^13^C incorporation into 2-OG [43]. Moreover, when 1 mM of 2-OG was added to NO_3_^−^ and NH_4_^+^ media, an increase in the *SOD Cl* and *SOD Ct* genes took place compared to NN, but not to NH_4_^+^ [50]. Thus, addition of 2-OG could play an important role in ROS scavenging.

**Figure 5:**
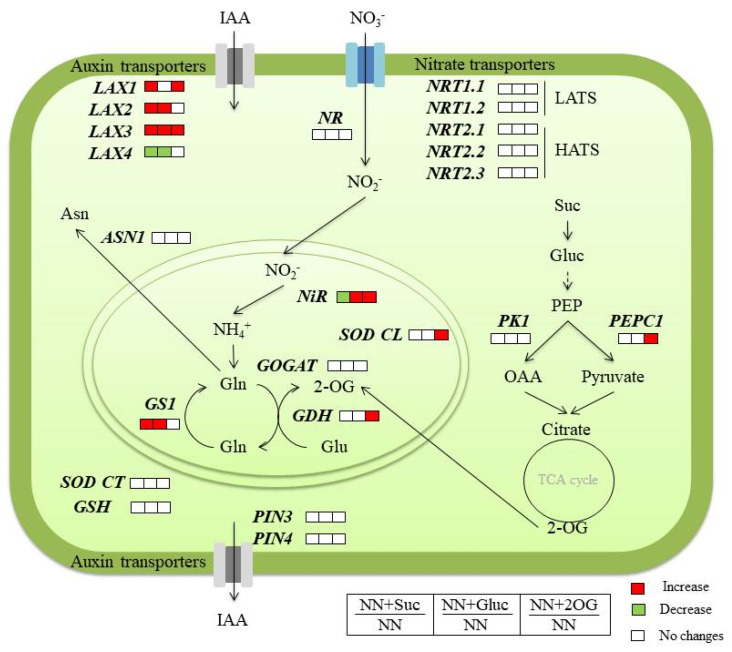
Schematic overview of the studied genes involved in N and C metabolism, auxin transport and antioxidant metabolism in tomato roots. Red cells show higher levels in plants with the treatment versus NN plants. Green cells showed lower levels in plants with the treatment versus NN plants (Tukey HSD test, *p* < 0.05).

To summarize, we investigated whether the addition of Suc or Gluc improved the root system architecture (RSA). This will allow us to examine in-depth nitrogen-use efficiency in the future, which is one of the approaches to take into account for cushioning the negative impact of climate change on plant growth and yield. Our results showed that sugar supply improves root development by enhancing auxin transporters *LAX1*, *LAX2* and *LAX3* gene expression (Figure 5). Furthermore, exogenous C supply modifies the expression of NO_3_^−^ assimilation genes. Taken together, these findings contribute to a better understanding about how these C sources can modulate N uptake and C/N, auxin and antioxidant gene expression. However, further studies are required to elucidate C-mediated responses in tomato seedlings and to extrapolate these results to field conditions.

## 4. Materials and Methods

### 4.1. Plant Materials and Growth Conditions

Tomato plants cv. Ailsa Craig seeds were sterilized with sodium hypochlorite (75% *v*/*v*) containing 0.1% of Tween 20 for 8 min. Then, they were washed with sterilized distilled water for 5 min and this step was repeated 4 times more. They were transferred to agar plates (1.5% *w*/*v*) and they were kept in darkness for 72 h in order to have a homogenous germination. Then, the homogeneously germinated seeds were placed on plates containing sterilized modified Hoagland solution medium composed of KNO_3_, Ca(NO_3_)_2_, MgSO_4_, H_3_BO_3_, H_3_PO_4_, ZnSO_4_, MoO_3_, CuSO_4_, MnSO_4_, sequestrene, agar (1.5% *w*/*v*) and MES buffer. The final N concentration was 10 mM and this media was considered as control treatment (NN medium; nitrate nutrition medium). Moreover, in order to test the effect of C supply, different C sources were added to the NN medium: sucrose (Suc), glucose (Gluc) and 2-oxoglutarate (2-OG). Suc has been added at 87 mM; Gluc at 55 mM and 2-OG at 0.27 mM (physiological levels). The physiological level concentrations were those detected in tomato roots grown under NO_3_^−^ (control) conditions in the previous study carried out by [67]. The pH of the different mediums was adjusted at 5.8–6.0. Then, seedlings were grown in the treatment plates for 7 days and plates were placed in a growth chamber at 26/18 °C of temperature (day/night) and 16/8 h photoperiod, maintaining the roots in darkness. Roots were collected, weighted and immediately placed in N_2_ liquid. Samples were stored at −80 °C for real time PCR analyses. The experiment was carried out with ten seedlings under each treatment in three independent replicates.

### 4.2. Root and Shoot Measurements

Primary root (PR) and shoot length and lateral root (LR) number were measured after 7 days of treatment and quantified via pictures with ImageJ software (National Institutes of Health, Maryland, USA). Root density was calculated as LR number divided by PR length. Fresh weight (FW) of roots and shoots was measured with the analytical balance Precisa 125A (Precisa, Dietikon, Switzerland). These measurements were carried out in at least 10 seedlings of each treatment of three independent replicates.

### 4.3. qRT-PCR Analyses

Gene expression was determined by means of Real Time-PCR. The RNA extraction of root tissue was done using RNeasy Plant Mini Kit (Qiagen, Hilden, Germany). For this, 1 μg of total RNA was digested with RNAase-free DNase (Promega, Wisconsin, USA) for 30 min at 37 °C and, after that time, RQ1 DNase stop buffer was added to the solution and incubated again for 10 min at 65 °C. Then, RNA was transformed into cDNA though the reverse transcription process according to PrimeScript RT kit instructions (Takara Bio Inc, Shiga, Japan) for 60 min at 37 °C. Finally, to run the Real Time PCR in the StepOne Real Time PCR System (Thermo Fisher Scientific, Massachusetts, USA), the total volume reaction was 10 μL and it was composed by 0.5 μL of forward and reverse primers, 5 μL of Sybrgreen qPCR (Thermoscientific Master Mix 2X reaction buffer; Thermo Fisher Scientific, Massachusetts, USA), 3 μL of RNase-free sterile water and 1 μL of sample cDNA. A list of the primers used in the qPCR is shown in Supplementary Table S1. Levels of *EF1α* gene expression were used as internal housekeeping control. The gene expression of NN grown seedlings were the same as showed by González-Hernández *et al*. [50].

### 4.4. Statistical Analyses

Statistical analyses were done using one-way analysis of variance in Statgraphics Centurion XVI.I software (Statistical Graphics Corp., Rockville, MD, USA). Results were expressed as means with standard errors and were compared using Tukey’s Honest Significant Difference (HSD) test with a 95% confidence interval (*p* < 0.05).

## Supplementary Materials

The following are available online at https://www.mdpi.com/2223-7747/9/7/837/s1, Table S1: Primer sequences [68,69,70].
