# Supplementary material for: Exogenous Carbon Compounds Modulate Tomato Root Development
Source: Plants (Basel). 2020 Jul 3;9(7):837. doi: 10.3390/plants9070837 (PMC7411956; doi:10.3390/plants9070837)
Supplement: Supplementary file 1 [file plants-09-00837-s001.zip › Table S1.pdf]

Table S1. Primer sequences

Gene	Genebank accession number	Forward	Reverse	Reference
EF1a	NM_001247106.1	GACAGGCGTTCAGGTAAGGA	GGGTATTCAGCAAAGGTCTC	[50]
NRT1.1	X92853	TACTATTCAAGCTATGGGTGTTACG	ATTTGTCCTCTTTCTTTTTTGTCCG	[69]
NRT1.2	X92852.1	CCTGAGACACAAAATGATGC	AGTCACCAAGTTTACAGCAA	[50]
NRT2.1	AF092705	TTCCTGTTACATTTTGTCATTTCCC	CAGATTCAAGACTATCCATTCCTCA	[69]
NRT2.2	AF092704	TCAAGGGAACGGAAGAACATTATTA	GCTCATTGAACTAAAGATTGACGAT	[69]
NRT2.3	AY038800	AATGCATGGTGTTACTGGTAGAGAG	CTAATAATAGGGACTAAAGGGGCTG	[69]
NR	X14060.1	TGGTTTCAATTGGGGTGCTG	ACTTTGAACCACCACCTCCA	[50]
NiR	AB211188.1	GCGTATATGCCTGCAACCAA	GTTTTCTGCCTGTTCCCTCG	[50]
GS1	AF200360.1	ATTGGCGGTTTTCCTGGCCC	ACTGTCCCGGCATGACTTCACC	[68]
GOGAT	XM_004234782.3	ACAGAGGTCAAAAGGCGAGT	CTTCCCTTGTGCTGCCATAC	[50]
GDH	U48695.1	AGCACGACAATGCACGAGGG	ATATTGGCGACCGCTGTCTTCC	[68]
ASN1	NM_001319849.1	TGCTCGATACTTGGCTGGAA	GCATCAATCCCGTCCTGAAC	[50]
PK1	SGN-U579698	AGAATGGGTTTAGGAGTAC	GTGGATCAAATGCACGAAC	[70]
PEPC1	AJ243416.1	TGTGAACCTGAACCCGACT	GTCCCCTATTCGGGACTTC	[70]
GSH	NM_001247085.2	CCTGGTGTTGATATGGTTCA	AAAGGATCAGCTTTTCTCGT	[50]
SOD Cl	NM_001247102.1	ACAGGACCACATTACAATCC	ACAACAGCTCTTCCAATGAT	[50]
SOD Ct	NM_001247840.1	ACCAGCACTACCAATTCTTT	AGTAAGGGGTTTAGGGGTAG	[50]
LAX1	NC_015446.2	CAGAAGCAAGCAGAGGAAGC	GAACCAAGCATCCCAAGCAG	
LAX2	NM_001247746.1	GTTTCGCTTGCACACCATTG	AACGGTGAAGCTAACGAGGA	
LAX3	HQ671070.1	TGAAGCACTCAGGACCAACA	AGCCCAATACACTGCACTTG	[50]
LAX4	NM_001247759.1	GTCTACTGGGCATTCGGAGA	CCAGACAAAGTACAAGGGCG	[50]
PIN3	NC_015441.2	TTCAAAATCAATTTAGCGTGTCA	CTCAAAATCCCTCTTGTTTCG	[50]
PIN4	NM_001247255.1	AAAGAGGGACCCACTGGACT	TCATAACACTAGCCGGAGGC	[50]
